# Thermophilin 13: In Silico Analysis Provides New Insight in Genes Involved in Bacteriocin Production

**DOI:** 10.3390/microorganisms11030611

**Published:** 2023-02-28

**Authors:** Francesco Salini, Lucilla Iacumin, Giuseppe Comi, Leon M. T. Dicks

**Affiliations:** 1 Department of Agriculture, Food, Environmental and Animal Science, University of Udine, 33100 Udine, Italy; 2 Department of Microbiology, Stellenbosch University, Stellenbosch 7600, South Africa

**Keywords:** thermophilin, *Streptococcus thermophilus*, gene organization, class IIb bacteriocin

## Abstract

Bacteriocins are a large family of ribosomally synthesised proteinaceous toxins that are produced by bacteria and archaea and have antimicrobial activity against closely related species to the producer strain. Antimicrobial proteinaceous compounds are associated with a wide range of applications, including as a pathogen inhibitor in food and medical use. Among the several lactic acid bacteria (LAB) commonly used in fresh and fermented food preservation, *Streptococcus thermophilus* is well known for its importance as a starter culture for yoghurt and cheese. Previous studies described the bacteriocin thermophilin 13 exclusively in *S. thermophilus* SFi13 and the genes encoding its production as an operon consisting of two genes (*thmA* and *thmB*). However, the majority of bacteriocins possess a complex production system, which involves several genes encoding dedicated proteins with relatively specific functions. Up to now, far too little attention has been paid to the genes involved in the synthesis, regulation and expression of thermophilin 13. The aim of the present study, using in silico gene mining, was to investigate the presence of a regulation system involved in thermophilin 13 production. Results revealed the dedicated putative bacteriocin gene cluster (PBGC), which shows high similarity with the class IIb bacteriocins genes. This newly revealed PBGC, which was also found within various strains of *Streptococcus thermophilus*, provides a new perspective and insights into understanding the mechanisms implicated in the production of thermophilin 13.

## 1. Introduction

*Streptococcus thermophilus* is a nonpathogenic lactic acid bacterium commonly isolated from bovine mammary tissue and raw milk, producing lactic acid, exopolysaccharides (EPS) and several organoleptic compounds from the fermentation of lactose and galactose, and also is a well-known starter culture used in the production of yoghurt and cheese [1]. The species has GRAS (Generally Regarded As Safe) status from the FDA (Food and Drug Administration) and QPS (Qualified Presumption of Safety) status from the EFSA (European Food Safety Authority). Several strains of *S. thermophilus* produce bacteriocins, which are small, ribosomally synthesized peptides with narrow or broad spectrum antimicrobial activity [2]. Examples include thermophilin A (strain ST134) [3], thermophilin T (strain ACA-DC 0040) [4], thermophilin 110 (strain 580) [5] and thermophilin 1277 (strain SBT1277) [6]. Two other unnamed bacteriocins were reported in *S. thermophilus* strain 81 and *S. thermophilus* strain 580, but little is known about their peptide sequence and genes encoding them [7,8].

Furthermore, apart from the mentioned examples, *S. thermophilus* SFi13, an isolate belonging to the Nestle’ strain collection, produces thermophilin 13. The inhibitory spectrum of thermophilin 13 includes *Clostridium botulinum*, *Listeria monocytogenes*, *Lactococcus lactis*, *Bacillus cereus*, *Bacillus subtilis* and *S. thermophilus* [9].

Expression of bacteriocin genes is usually subject to external induction factors (IFs) regulation. The gene encoding the pre-peptide is normally located in the same operon as genes encoding the immunity protein, ABC transporter and accessory protein [10]. The accessory protein may also be involved in rendering immunity to the bacteriocin-producing cell [11]. The cleavage site that characterises peptides ThmA and ThmB is preceded by a double-glycine motif found in pre-peptides of class IIb bacteriocins [12,13]. However, thermophilin 13 has been described as an atypical bacteriocin in the sense that the activity of the antibacterial peptide ThmA is enhanced by the peptide ThmB, encoded by genes *thmA* and *thmB*, respectively, on a 960-bp operon (U93029.1) [9]. Thermophilin 13, in agreement with the classification proposed by Zouhir et al. (2010), shares common characteristics with class IIe bacteriocins by having a WX9GX3G motif (1.02 × 10^−7^ < *p*-value < 7.01 × 10^−6^) in the enhancer peptide ThmB. However, the YGNGV-C motif is missing in both the peptides ThmA and ThmB. The YGNGV-C motif is typical of the anti-*Listeria*-active peptides [13]. Marciset et al. (1997) described thermophilin 13 as an ionophoric poration complex formed by the interaction between ThmA and ThmB [9]. Their results did not provide any information about the involvement of genes other than *thmA* and *thmB* forming the operon, which regulates the production of thermophilin 13. Among bacteriocin-producer strains, lactic acid bacteria play a key role in fresh and fermented food preservation. The present study is focused on *Streptococcus thermophilus* SFi13 strain, which is the only producer reported in the scientific literature of the bacteriocin thermophilin 13. Based on current knowledge, only two genes, *thmA* and *thmB,* are involved in the thermophilin 13 productions, and to our knowledge, no further studies have been pursued to investigate this bacteriocin’s mode of action and gene organisation. Therefore, our study aimed to investigate the identity and organisation of all the genes encoding proteins involved in thermophilin 13 regulation, synthesis, transport and immunity, using in silico DNA comparisons.

## 2. Materials and Methods

### 2.1. Genome Sequences

The thermophilin 13 operon sequence (U93029.1) amounting to 960-bp and listed in the National Center for Biotechnology (NCBI, Bethesda, MD, USA; https://www.ncbi.nlm.nih.gov/; accessed on 2 October 2022) nucleotide database was used to conduct a similarity search using the NCBI Basic Local Alignment Search Tool (BLAST) [14]. Similarities to the thermophilin 13 operon were determined using NCBI Sequence Viewer [15]. The complete genome sequences of all bacterial strains showing the presence of 960-bp with an identity of 100% with the thermophilin 13 operon (U93029.1) sequence were downloaded from the NCBI database.

### 2.2. Identification and Analysis of the Thermophilin 13 Biosynthetic Gene Cluster (BGC)

To identify potential bacteriocins, biosynthetic gene clusters (BGC) of all genome sequences were analysed using the command-line antiSMASH version 5.0 [16] and BAGEL4 [17]. The ClusterFinder algorithm with additive cluster discovery was used. ClusterFinder source code is available from the GitHub repository (https://github.com/petercim/ClusterFinder; accessed on 2 October 2022).

Amino acid sequences with predicted ORFs (open reading frames) were compared against the non-redundant protein database using Blastp version 2.9.0+ (protein–protein BLAST) [18]. Using the Jukes–Cantor model, a Nearest-Neighbor-Interchange (NNI) tree with 1000 Bootstraps was constructed, including all bacteriocin gene sequences provided by antiSMASH and BAGEL4 hosted on the NCBI website. Analyses were conducted using the MEGA 11 software (Version 11.0.11) platform [19].

Putative bacteriocin genes within the respective genomes were annotated using the CLC Main Workbench—QIAGEN Bioinformatics software (CLC bio, Aarhus, Denmark). Sequence alignment was performed using Muscle WS [20] and displayed by Tree Of Life (iTOL) v4 online tool [21]. Comparative analyses of genomic datasets were performed using Operon-mapper [22] and loci of selected bacteriocin genes were visualised using cblaster (github.com/gamcil/clustermap.js; accessed on 16 November 2022) [23], Clinker & Clustermap.js (github.com/gamcil/clinker; accessed on 16 November 2022) [24] and protein 3D prediction was obtained with ColabFold open-source software available at https://github.com/sokrypton/ColabFold; accessed on 15 February 2023 [25].

## 3. Results

Currently, no complete genome sequence of *S. thermophilus* SFi13 is available on the NCBI database. In a study by Comelli et al. (2002) and in the deposited patent USOO7491386B2, the authors described and evaluated bacterial strains with potential properties as oral probiotics, useful for the prevention of dental caries. According to the Nestlé Culture Collection (NCC), they also affirmed that strain *S. thermophilus* SFi13 was reclassified as *S. thermophilus* NCC 2008 [26,27].

All prior research on this bacteriocin only refers to the partial sequence with Accession Number U93029.1 (NCBI). Despite the reclassification of strain S. *thermophilus* SFi13 to *S. thermophilus* NCC 2008, the genome sequence is also unavailable on the NCBI database. The DNA sequences of operon U93029.1 contained genes *thmA* and *thmB*, putative promoter elements, ribosome binding sites, and a rho-independent terminator structure, as reported by Marciset et al. (1997) [9]. A similarity search using BLAST identified S. *thermophilus* B59671 (CP022547.1), *S. thermophilus* KLDS 3.1003 (CP016877.1), *S. thermophilus* STH_CIRM_1049 (LR822034.1), *S. thermophilus* STH_CIRM_1048 (LR822033.1), *S. thermophilus* CS9 (CP030927.1), *S. thermophilus* DMST-H2 (CP063275.1), TK-P3A (CP045596.1), ATCC 19258 (CP038020.1), *S. thermophilus* LMD-9 (CP086001.1), *S. thermophilus* NCTC12958 (LS483339), and *Streptococcus macedonicus* 19AS (PEBN00000000.1) shares identical DNA sequences to the thermophilin 13 operon (U93029.1) of *S. thermophilus* SFi13. All these strains have 100% similarity and 100% identity with the operon U93029.1. Even though some sequences had 79.22–84% of identity with operon U93029.1, their query cover ranged from 8–14%, and for this reason, they were automatically excluded from this study. General information and identification code of these 11 strains are listed in Table 1.

**Figure 1:**
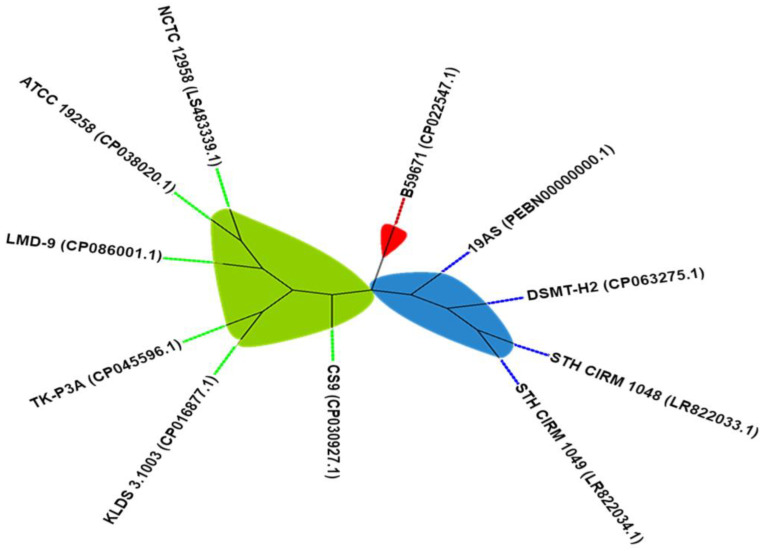
Cladogram tree showing the phylogenetic relatedness amongst gene loci, including thermophilin 13 (U93029.1). The tree was composed using the maximum likelihood method but visualised by removing branch length information. The three nodes are shown in different colours.

Data obtained using BAGEL4 and antiSMASH version 5.0 confirmed the distribution of thermophilin 13 BGC (biosynthetic gene cluster) in all 11 strains of *S. thermophilus*. All strains showed the same area of interest (AOI), with some variation in nucleotide sequences. Strains *Streptococcus thermophilus* B59671 (CP022547.1) and *Streptococcus macedonicus* 19AS (PEBN00000000.1) were the most diverse based on AOI. Figure 1 shows a cladogram tree that is derived from the multiple sequence alignments of AOIs identified from the in silico study.

Known bacteriocin loci were detected, e.g., the lantibiotic salivaricin 9 operon in the genome of *S. thermophilus* NCTC 12958 and *Streptococcus thermophilus* ATCC 19258 and thermophilin 110 operon in *S. thermophilus* B59671 [5]. The entire locus of Salivaricin 9 was fully characterized from *S. salivarius* strain JIM8780, and it was shown to consist of eight genes, having the following putative functions: sivK, sensor kinase; sivR, response regulator; sivA, Sal9 precursor peptide; sivM, lantibiotic modification enzyme; sivT, ABC transporter involved in the export of Sal9 and concomitant cleavage of its leader peptide; and sivFEG, encoding lantibiotic self-immunity [39]. The broad-spectrum bacteriocin thermophilin 110 is encoded within the blp gene cluster. Furthermore, thermophilin 110 was reported to inhibit the growth of *Listeria monocytogenes*, *Streptococcus mutans*, *Streptococcus pyogenes* and *Propionibacterium acnes*.

**Figure 2:**
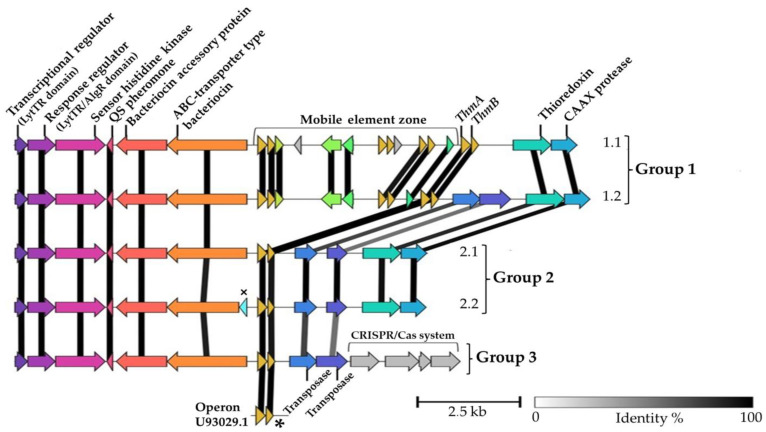
Comparison of the BGCs of thermophilin 13. Predicted ORFs are represented as arrows. Three main gene arrangements were detected. Subgroup 1.1 contains strain *S. macedonicus* 19AS and subgroup 1.2 strains *S.thermophilus* STH_CIRM_1049, *S. thermophilus* STH_CIRM_1048, *S. thermophilus* DMST-H2. Group 2 is divided into two subgroups, with strains *S. thermophilus* KLDS 3.1003, S. thermophilus TK-P3A, *S. thermophilus* CS9 in subgroup 2.1 and strains *S. thermophilus* LMD-9, *S. thermophilus* ATCC 19258 and *S. thermophilus* NCTC 12958 in subgroup 2.2. Group 3 is represented by strain *S.thermophilus* CS9. The symbol * indicates the third ORF (ORFC), encoded by the U93029.1 operon and symbol × indicate the C39 peptidase-like domains found only in subgroup 2.2. Colours are based on ORFs similarity found, including the mobile element zone, which were identified as small ORFs with apparent unrelated functions in the bacteriocins productions due to the prediction as hypothetical proteins.

Manual curation and annotation were performed to compare the differences between the ORFs predicted by the bacteriocin mining tools. Comparisons of AOIs indicated that the gene loci in the thermophilin 13 operon are organised into eight genes/ORFs encoding proteins related to bacteriocin production, plus the two thermophilin 13 structural genes. These were consistent for all strains and include a response regulator (RR), sensor histidine protein kinase (HPK), quorum-sensing system pheromone BlpC, ABC-transporter, bacteriocin accessory protein, thiol–disulfide oxidoreductases, CAAX protease and genes *thmA* and *thmB*. Despite the similarity in translation, three operon patterns were observed using the Operon-mapper web server. These variations, including all strains analysed in the present study, are schematically visualised in Figure 2.

A similar regulation and secretion system was observed for thermophilin 13 in groups 1, 2 and 3 (Figure 2). However, an additional nucleotide sequence (mobile element zone) was detected in group 1 (Figure 2). No variations in gene transcription upstream and downstream of this area were observed. In this regard, the insertion element, which is present in all sequenced BGCs of cluster 1, requires further investigation to assess possible interference with thermophilin 13 production due to the presence of transposases. A third ORF (ORFC), encoded by the U93029.1 operon, was reported by Marciset et al. (1997) [9] and was found in all BGCs groups shown in Figure 2. Structure models of the poration complex formed by Thermophilin 13 were described as the ThmA enhancing ThmB peptide with maximal explication in antimicrobial activity in equimolar concentration. However, the peptide ThmA alone resulted in antibacterial activity against *S. thermophilus*, *Clostridium botulinum*, *Listeria. monocytogenes* and *Bacillus cereus*.

**Figure 3:**
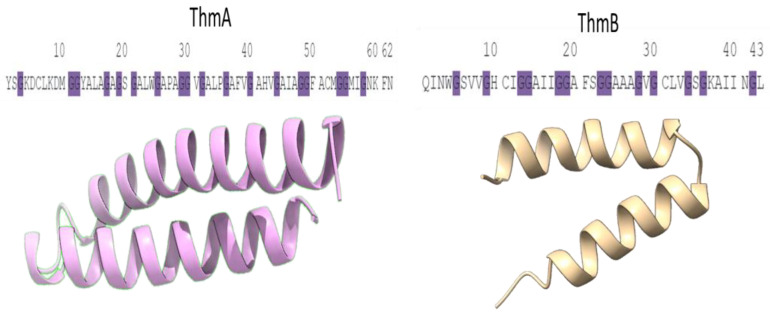
Amino acid sequences of the unmodified two-peptide subunit of thermophilin 13. The glycine residues in both peptides are matched in purple.

In this regard, the presence of GxxxG-motifs or GxxxG-like motifs AxxxA and SxxxS motif, instead of the GxxxG-motif and a high helical content were related to the two-peptide bacteriocins into form membrane-penetrating helix–helix structures, explaining the increased helical content forming a dimer complex, in which an incremented antimicrobial action is attributable [40] This dual peptide interaction was described in several class IIb bacteriocins including thermophilin 13 as is reported by the authors Oppegård et al. (2008) and Nissen-Meyer et al. (2010) [41,42]. However, this aspect requires further investigation due to multiple GxxxG motifs, located in positions ^21^GxxxG^25^, ^32^GxxxG^36^, ^40^GxxxG^44^, ^54^GxxxG^58^ for ThmA and ^5^GxxxG^9^, ^14^GxxxG^18^, ^15^GxxxG^19^, ^19^GxxxG^23^, ^24^GxxxG^28^ for ThmB peptides, respectively, as is showed in Figure 3.

## 4. Discussion

Most bacteriocin operons include genes involved in the post-transcriptional modification and/or secretion of these peptides [12]. Based on that, the present study examined the thermophilin 13 operon (U93029.1) described by Marciset et al. (1997) [9], which appears lacking in bacteriocin-regulating genes involved in bacteriocin synthesis.

In silico analysis is an excellent predictor of “bacteriocin-associated driver genes” within genomes genes adding information on the mechanism related to the specific bacteriocin production. Starting from genomic or amino acid sequences, the main advantages of these methods are a significant reduction in time in comparison to the traditional screening method and, subsequently, the costs embroiled to the use of laboratory materials. Antimicrobial genome-mining tools have been closing the gap between a large number of predicted biosynthetic gene clusters (BGC) encoding bacteriocins, including ribosomally synthesized, post-translationally modified peptides (RiPPs) and also polyketide synthases (PKS) and non-ribosomal peptide synthetases (NRPS) [43]. However, the presence of bacteriocin genes in a strain is always directly related to an effective translation into biological antimicrobial activity [44].

This comprehensive in silico study reveals a complete thermophilin 13 gene cluster containing genes encoding a response regulator (RR), sensor histidine protein kinase (HPK), quorum-sensing system pheromone BlpC, ABC-transporter, bacteriocin accessory protein, thiol–disulfide oxidoreductases, CAAX protease and genes *thmA* and *thmB*.

Furthermore, we confirm the presence of ORFC in all strains; however, no correspondence related to this peptide has been associated with bacteriocin production in the databases.

Similarly to other bacteriocin gene clusters, response regulators grouped as LytR/AlgR family (RR) were predicted [45]. These regulators explicate their function in binding to promoters that initiate the transcription after phosphorylation of Asp residues promoting bacteriocin production and autoactivating their respective operons [46]. LytR Regulatory Systems represents the most abundant type of transcriptional regulator in the prokaryotic kingdom involved as either activators or repressors of single or operonic genes; of genes, including those involved in virulence, metabolism, quorum sensing motility and bacteriocins [45,47]. Furthermore, histidine kinases and response regulators mediate the actual response regarding bacteriocin production by a two-component signal-transducing system [48,49,50,51].

Peptide MTKHRTSLTAFTELSPSELHRISGGDWWDWMKYFPSKQAIDSNKHKLG is present in all groups. By identifying the potential role in the bacteriocins biosynthetic gene cluster of this peptide, the prediction showed affinity to the quorum-sensing pheromone BlpC (PF03047 HMM), which is also appointed as ComC/BlpC family leader-containing pheromone/bacteriocin. Interestingly, these peptides are different but are reported in several quorum-sensing regulated bacteriocins in *S. thermophilus*, stimulating the production of BLP (bacteriocin-like peptides) as a signal peptide for the activation of bacteriocin synthesis through a three-component regulatory system consisting of a peptide pheromone, a membrane-associated histidine protein kinase, and response regulators [52,53]. Plantaricins A, E/F and J/K by *L. plantarum* of C11, sakacin A of *L. sakei* Lb706EF and sakacin P of *L. sakei* LTH673102 are the best examples of bacteriocin of class II regulated by the three-component regulatory system, including inducing peptide (an indicator of the cell density), which is sensed by the corresponding (HPK), resulting in the activation of the RR [54].

A dedicated bacteriocin ABC-transporter, including a peptidase C39 motif, predicted to be a bacteriocin/lantibiotic transporter based on conserved domains (COG227400), and a bacteriocin accessory protein generally associated with transport, was observed [55,56]. ABC-transporter proteins related to the class II bacteriocin maturation and secretion carry a proteolytic peptidase C39 domain in their N-termini. The proteolytic peptidase C39 cleaves a double glycine (GG) motif-containing signal peptide from substrates before secretion, modulated in association with an ATP-binding cassette component located in the same protein [57,58]. Differences in ABC transporter sequences in Group 2 were detected in the C39 motif. Interestingly, an independent protein containing C39 peptidase domains, in terms of amino acid sequences, is present in subgroup 2.2. This protein conformation is termed C39 peptidase-like domains (CLD); additionally, their role is not yet completely understood, and they appear degenerated with nonproteolytic activity [59,60]. Most endopeptidases of family C39 are the less conservative component in the entire bifunctional transporter protein with a dedicated catalytic function for the secretion of the antimicrobial peptide of interest [61].

Thiol–disulfide oxidoreductases (TDORs) in Gram-positive bacteria play an essential role in forming disulfide bonds, allowing correct folding in class II bacteriocins through the R–S–S–R′ bond of the CXXC catalytic site resulting in disulfide-bonded cysteines [62,63]. In this regard, only thmA has two cysteine residues in positions 6 and 53 of the aminoacidic backbones. The aminoacid methionine and single cysteines are also vulnerable to oxidation, but it has never been reported the disulfide bridge formation with this conformation in bacteriocins. However, in this protein, the LPxTG motif membrane-anchored transpeptidase, which cleaves proteins between the threonine (Thr) and the glycine (Gly), is conserved.

Interestingly, ThmA and ThmB peptides lack Thr residues; this is in accordance with Marciset et al. (1997), who observed that the oxidation of methionines to methoxides in position (Met^10^, Met^54^ and/or Met^57)^ of ThmA seems the only possible explanation of the proposed poration complexes (AB)n (i.e., Thermophilin 13) [9].

CAAX metalloproprotease (bacteriocin-processing enzymes) detected in bacteriocin loci, including the Abi genes downstream of the bacteriocin structural genes, is likely involved in self-immunity. The role of these conserved motifs in the immunity function conferred a high degree of cross-resistance against each other’s bacteriocins, suggesting the recognition of a common receptor. An example of this mechanism was found in *Latilactobacillus sakei* 23K [64]. Furthermore, the bacteriocin-like gene sak23Kalphabeta showed antimicrobial activity when expressed in a heterologous host, and the associated Abi gene sak23Ki conferred immunity against the related bacteriocin [65,66]. Genes encoding the production, secretion, regulation, and immunity of thermophilin 13 are similar to gene sequences reported for class II bacteriocins from *S. thermophilus* strains LMG18311, CNRZ1066, and LMD-9 [67]. However, in *S. thermophilus* B59671, belonging to Group 3, TDOR and CAAX protease are replaced with a CRISPR/Cas system. Prior studies have noted the importance of quorum sensing induction peptides encoded by the different blp gene clusters found in *S. thermophilus* strains ST109, LMD-9, ST106, LMG18311, CNRZ1066, ND03, JIM8232, MN-ZLW002 and B59671 due to their homology to a bacteriocin-like peptide (blp) gene cluster in *S. pneumoniae* [28,51,68,69]. In relation to this aspect, the strains *S. thermophilus* B59671, ST106, ST109 and LMD-9 have been shown to produce a broad spectrum of bacteriocins encoded within a bacteriocin-like peptide (blp) gene cluster. However, the thermophilin 13 operon is also present in LMD-9 and B59671 strains but must not be confused with the bacteriocin-like peptide (blp) in *S. pneumoniae* gene clusters mentioned above. In this regard, strains LMD-9 and B59671 could be multiple-bacteriocins producer strains and should be highlighted for the necessary evaluation of the role of environmental factors and medium composition on bacteriocin production.

Bacteriocin production is an energy-utilising process involving a cascade of genetic mechanisms that varies greatly in how bacteriocin loci are organised. Among bacteriocin production mechanisms, in many strains, quorum-sensing (QS) circuits modulate various physiological responses, including the production of antimicrobial compounds [70,71]. However, in silico screens can be limited by their dependence on similarity to those previously described by Walsh et al. (2015) [72]. Further work is required to confirm that operon variation between strains influences the production of thermophilin 13. In summary, these results highlight that the production of peptides ThmA and ThmB is strongly related to its PBGC, which is not limited to only *thmA* and *thmB* genes. Therefore, it can be assumed that the QS system regulates the expression of thermophilin 13 bacteriocins in several *S. thermophilus* strains. It has to be considered that since 1997 no other investigations have been made on this bacteriocin. However, the evidence gathered in this study provides further insights into the mechanism of production and regulation of thermophilin 13; this has been observed and described to the scientific community after twenty-five years. All these reported strains are used in industrial applications, and their technological properties have already been proven, opening a new panorama of research that need further investigation. In light of the urgent need for new weapons to counteract pathogens without the use of antibiotics, the identification of the most suitable thermophilin 13 producer strain in terms of bacteriocin production and its applicability in food manufacturing is relevant.

## 5. Conclusions

The significance of antimicrobial peptides (AMPs) is growing for applicability in various fields, including as a bioprotector agent. There are still many challenges regarding bacteriocins looking for an answer, such as structural multiplicity, different modes of action, different classes, and the high cost of production. Furthermore, also for bacteriocins already applied as preservative agents, the major issues are connected to finding strategies for optimizing their maximum rate of production and developing more effective purification steps from the bacterial supernatant, which are currently long and complicated.

A large number of genomes available in public repositories offer novel approaches valuable in identifying novel bacteriocin genes and gene clusters [54]. Screening of putative bacteriocin gene clusters provides a deeper understanding of how these peptides are regulated. Genome mining indicates that operon thermophilin 13 is present in several strains grouped in three different clusters on the basis of the different genes organization of the eight genes involved in these bacteriocins’ biosynthesis. As Marciset et al. (1997) suggested in their conclusion, thermophilin 13 showed peculiar and different characteristics in its mode of action that can share functional properties of lantibiotics. Our results also indicated that the thermophilin 13 two-component peptide system belongs to class IIb with its own related genes cluster, composed of a response regulator (RR), sensor histidine protein kinase (HPK), quorum-sensing system pheromone BlpC, ABC-transporter, bacteriocin accessory protein, thiol–disulfide oxidoreductases, CAAX protease and genes *thmA* and *thmB.* However, the predictions obtained from the present research and the others in silico studies in general, must not be accepted as conclusive evidence for bacteriocin production, and we do not claim that all strains included in this study can produce thermophilin 13 in vitro and/or in vivo. Nevertheless, the information obtained in this study shed some light on the possible quorum sensing involvement in the mechanisms of regulation and secretion of thermophilin 13, which has already been reported in *Streptococcus thermophilus* strains having bacteriocin-like peptide (blp) gene cluster. This is a solid starting point for further investigation into a topic that has not been explored since 1997, which provides opportunities to expand the knowledge of this antimicrobial peptide in order to target effective applications for food safety.

## Figures and Tables

**Table 1:** List of strains and associated GenBank accessions code for genomes in which a 960-bp sequence with 100% identity to U93029.1 was found using BLASTn.

Strains	GenBank Code	Isolation/Source	Reference
*Streptococcus thermophilus* B59671	CP022547.1	Milk	[28]
*Streptococcus thermophilus* KLDS 3.1003	CP016877.1	Lactic starter (for yoghurt production)	[29]
*Streptococcus thermophilus* LMD-9	CP086001.1	Lactic starter (for yoghurt and mozzarella production)	[30]
*Streptococcus macedonicus* 19AS	PEBN00000000.1 ***	Cheese	[31]
*Streptococcus thermophilus* STH_CIRM_1049	LR822034.1	Lactic starter (for yoghurt production)	[32,33] **
*Streptococcus thermophilus* STH_CIRM_1048	LR822033.1	Lactic starter (for yoghurt production)	[32,33] **
*Streptococcus thermophilus* CS9	CP030927.1	Fermented milk	[34]
*Streptococcus thermophilus* DMST-H2	CP063275.1	Probiotic products	[35]
*Streptococcus thermophilus* TK-P3A	CP045596.1	Pasteurised milk	[36]
*Streptococcus thermophilus* ATCC 19258 *	CP038020.1	Milk	[37]
*Streptococcus thermophilus* NCTC 12958 *	LS483339.1	Milk	[38]

* Genome sequences of *S. thermophilus* NCTC 12958 and *S. thermophilus* ATCC 19258 are identical. In this study, both genomes were analysed independently. ** The identification name of the strains of *Streptococcus thermophilus* STH_CIRM_1048 and *Streptococcus thermophilus* STH_CIRM_1049 is related to the accession codes for genomes valid for the NCBI database; the same strains are reported as *Streptococcus thermophilus* CIRM-BIA1048 *Streptococcus thermophilus* CIRM-BIA1049 in citation [32,33]. *** GenBank code PEBN01000000.1 and PEBN01000052.1 both refer to *Streptococcus macedonicus* 19AS strain in NCBI database.

## Data Availability

All Sequences are available in the NCBI database with related GenBank code reported in Table 1. The names of the repository/repositories and relative literature can be found in the article. Curated Putative Bacteriocin Gene Clusters (PBGCs) described in this study are readily available upon reasonable request to the corresponding author.
